# The Effects of Adding Transcutaneous Spinal Cord Stimulation (tSCS) to Sit-To-Stand Training in People with Spinal Cord Injury: A Pilot Study

**DOI:** 10.3390/jcm9092765

**Published:** 2020-08-26

**Authors:** Yazi Al’joboori, Sarah J. Massey, Sarah L. Knight, Nick de N. Donaldson, Lynsey D. Duffell

**Affiliations:** 1 Department of Medical Physics & Biomedical Engineering, UCL, London WC1E 6BT, UK; n.donaldson@ucl.ac.uk (N.d.N.D.); l.duffell@ucl.ac.uk (L.D.D.); 2 Aspire CREATe, UCL, Stanmore HA7 4LP, UK; sarah.massey.13@ucl.ac.uk; 3 London Spinal Cord Injury Centre, Royal National Orthopaedic Hospital, Stanmore HA7 4LP, UK; sarah.knight23@nhs.net

**Keywords:** human, neuromodulation, neurorehabilitation, non-invasive, spinal cord injury, transcutaneous spinal cord stimulation

## Abstract

Spinal cord stimulation may enable recovery of volitional motor control in people with chronic Spinal Cord Injury (SCI). In this study we explored the effects of adding SCS, applied transcutaneously (tSCS) at vertebral levels T10/11, to a sit-to-stand training intervention in people with motor complete and incomplete SCI. Nine people with chronic SCI (six motor complete; three motor incomplete) participated in an 8-week intervention, incorporating three training sessions per week. Participants received either tSCS combined with sit-to-stand training (STIM) or sit-to-stand training alone (NON-STIM). Outcome measures were carried out before and after the intervention. Seven participants completed the intervention (STIM N = 5; NON-STIM N = 2). Post training, improvements in International Standards for Neurological Classification of Spinal Cord Injury (ISNCSCI) motor scores were noted in three STIM participants (range 1.0–7.0), with no change in NON-STIM participants. Recovery of volitional lower limb muscle activity and/or movement (with tSCS off) was noted in three STIM participants. Unassisted standing was not achieved in any participant, although standing with minimal assistance was achieved in one STIM participant. This pilot study has shown that the recruitment of participants, intervention and outcome measures were all feasible in this study design. However, some modifications are recommended for a larger trial.

## 1. Introduction

Spinal cord injury (SCI) is a life-long condition, which can substantially impact the health and well-being of affected individuals. Appropriate management is essential to maximise health-related quality of life. Standing remains one of the only clinical interventions used both acutely and following discharge from hospital, in people with chronic SCI, because it is affordable and relatively simple to do at home. Regular standing has many known health benefits, including reduced muscle tone, improved blood flow in the lower limbs, beneficial effects on bladder and bowel function and improvements in quality of life [1]. Active stand training, with additional electrical stimulation, may facilitate independent or minimally-assisted standing in people with chronic SCI [2].

Non-patterned spinal cord stimulation (SCS), delivered by electrodes implanted in the epidural space of the spinal cord has been shown to elicit lower limb extensor movements in rats [3,4] and humans [5,6,7] with motor complete SCI, via activation of large-to-medium diameter sensory fibres within the posterior roots (below the level of the injury). Transcutaneous SCS (tSCS), which uses non-invasive electrodes placed over the T12—L1 vertebrae and abdomen, has been shown, by neurophysiological [8] and computer simulation [9] studies, to recruit similar neural structures as epidural SCS. Single pulses of tSCS elicited compound muscle action potentials in lower limb muscles, due to transynaptic activation of α-motoneurons, termed Posterior Root Reflexes (PRRs), which are susceptible to homosynaptic depression when paired pulses are applied [10,11,12]. Similar to epidural SCS, supra-threshold tSCS has been shown to augment or generate lower limb extension in people with motor complete and incomplete SCI [13,14]. This delivery method removes the requirement for surgery and is simple to apply, so it may be readily transferable for clinical use; however, it can cause some discomfort and unwanted muscle contractions as the current passes through skin and trunk musculature. Transcutaneous SCS also has less specificity than epidural SCS and may be more affected by changes in body position [15].

When delivered at an intensity below that required to produce motor response (i.e., sub-threshold), epidural SCS has been shown to enable individuals with motor complete SCI to produce volitional movement in their otherwise paralysed muscles [16,17,18], and to augment volitional standing and stepping [17,19,20,21], offering an important therapeutic pathway for people living with chronic SCI [22]. Transcutaneous SCS, delivered at an intensity that is sub-threshold for generating lower limb activity, either muscle contractions or whole-limb responses, but high enough to produce paraesthesia in lower limb dermatomes, has also been shown to augment volitional stepping on a treadmill [23,24], and to suppress lower limb spasticity [25,26,27] in people with incomplete SCI.

After several months of rehabilitative training combined with epidural SCS, progressive improvements in volitional movements, standing and stepping (in the presence of SCS) have been reported in pre-clinical [28,29,30] and clinical [16,17,19,31,32] studies. Furthermore, it has recently been reported that this recovery of volitional motor control after SCS training was possible even when the SCS was switched off, indicative of neuroplastic recovery. This was achieved in people with incomplete SCI after several months of epidural SCS combined with intensive locomotor training [33], and in people with motor complete injuries after one month of daily epidural SCS, used to achieve volitional movement and specific autonomic functions (such as blood pressure regulation) [34].

The only studies to report progressive improvements in the generation of lower limb activity in the presence of non-invasive tSCS [14,35] used a specific stimulation waveform for tSCS (each pulse filled by a 10 kHz carrier frequency [36]); this is thought to reduce the discomfort associated with tSCS. With this waveform, supra-threshold tSCS has been used to achieve minimally-assisted standing in people with motor complete and incomplete injuries, and progressive improvements in standing were reported after several weeks of training [14]. In the current study, we explored the effects of sub-threshold tSCS combined with sit-to-stand training on recovery of motor control in people with complete and incomplete SCI, using a conventional stimulation waveform. This was compared to sit-to-stand training alone, in order to specifically explore the importance of additional tSCS to achieve motor recovery. We also measured the effects on health-related quality of life and functional independence.

The main aim of this pilot study was to explore the feasibility of comparing a sit-to-stand training intervention, with and without additional sub-threshold tSCS, in people with motor complete and incomplete SCI. We found that people with both complete and incomplete injuries were willing to take part in the trial, however participants were more willing to participate in the tSCS group than sit-to-stand training alone. In this small group of participants, we found increases in volitional activation of specific lower limb muscles after training, only in the participants that received tSCS.

## 2. Experimental Section

This trial had ethical approval from the London–Stanmore Research Ethics Committee (REC reference: 18/LO/0784) and all participants provided written informed consent to take part. The trial was registered with ClinicalTrials.gov (NCT03536338).

### 2.1. Participants

Participants were recruited from the London Spinal Cord Injury Centre (Royal National Orthopaedic Hospital) and Neurokinex (specialised neurological activity-based rehabilitation facilities). Inclusion criteria were (1) spinal cord injury for >1 year, (2) SCI level C5-T12, (3) aged >18 years, (4) AIS A-D, (5) unable to stand from a chair unaided. Exclusion criteria were (1) cardiac pacemaker (2) any other musculoskeletal diagnosis affecting the lower limbs, (3) pregnancy, (4) complex regional pain syndrome, (5) implanted metal or active device at electrodes caudal to T9 (e.g., screws, contraceptive coil), (6) spinal malignancy, (7) uncontrolled autonomic dysreflexia, (8) neurological degenerative diseases, (9) peripheral nerve damage affecting the lower limbs, (10) currently on any form of anti-spasticity treatment (e.g., Botox, but not including bladder Botox), (11) osteoporotic-bone density T-score less than −2.5. The demographics and injury characteristics of the 9 participants assessed at baseline are provided in Table 1.

### 2.2. Study Design

**Figure 1:**
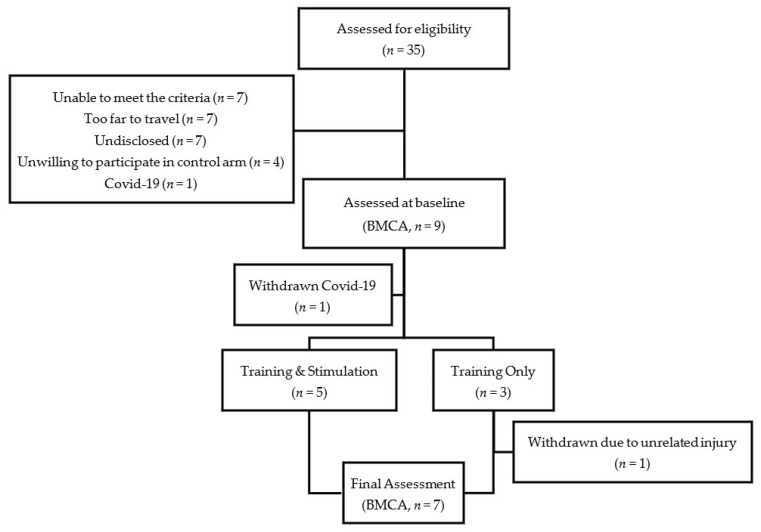
CONSORT flow diagram.

This was a purposefully sampled cohort study, designed to balance Training + tSCS (STIM) and Training Only (NON-STIM) groups by AIS grade. Participants were initially assessed against the inclusion/exclusion criteria, and then assigned to either the STIM or NON-STIM group: we aimed to recruit at least two participants in each group with motor complete injuries (AIS A/B), and two participants in each group with incomplete injuries (AIS C/D). Thirty-five participants were assessed for eligibility; of these, 26 did not participate in the trial. Of these, applicants unable to meet criteria (*n* = 7) was primarily due to existing implanted metal work being too low and applicants being less than 1-year post-injury. Others expressed that the training center locations were too far to travel 3 times a week for 8 weeks (*n* = 7), that they were unwilling to participate in the study after being assigned to the control arm (*n* = 4), or did not disclose the reason (*n* = 7). One applicant was willing to participate, but unable to start the study due to early termination (due to the COVID-19 pandemic). Therefore, nine participants were assessed at baseline; of these, 7 completed the intervention and 2 were withdrawn due to a lower limb injury (*n* = 1) or early termination (*n* = 1). See CONSORT flow diagram (Figure 1).

### 2.3. Intervention

All training was carried out at Neurokinex and consisted of 24 sessions (3 sessions per week for 8 weeks). During each session, participants transferred to a physiotherapy couch and were fitted with a body weight support (BWS) harness. The harness was attached to a Keiser Power Rack (Keiser UK Ltd., Gloucestershire, UK), which incorporates a pneumatic resistance system to assist the participant in the sit-to-stand manoeuvre by partially supporting their bodyweight. During each training session, participants stood up 5 times, taking approximately one-hour. Each sit-to-stand manoeuvre was initiated by increasing the BWS to assist the participant into stand; two therapists were available to stabilise the participant if required. Standing was then maintained for 4–5 min, during which postural exercises such as deep and shallow squats, lateral and anterior/posterior weight shifts, squat holds, single leg bends, hip thrusts, kettle bell arm presses, trunk strengthening (arms off bar and straighten posture), hip rotations and squat rotations were performed. The participants were then returned to a seated position and rested for 2–3 min before the cycle was repeated. In the STIM group only, continuous tSCS was applied during active standing (sit-to-stand, standing and sit-from-stand), no tSCS was provided during seated rest.

### 2.4. Stimulation Parameters

**Figure 2:**
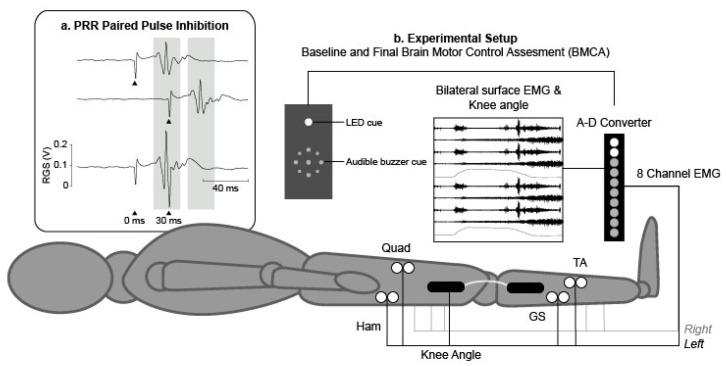
(**a**) Example traces during posterior root reflex (PRR) testing; single pulses were delivered at 0 and 30 ms (upper traces) and as a pair with an interstimulus interval (ISI) of 30 ms (lower trace) to demonstrate paired pulse inhibition (arrows denote the time at which the stimulus was applied). (**b**) Experimental setup for baseline and final Brain Motor Control Assessments. Participants were placed in a supine position with bilateral electromyography (EMG) electrodes placed over the Quadriceps (Quad), Hamstring (Ham), Tibialis Anterior (TA) and Gastrocnemius (GS) muscles to record EMG and electro-goniometers were placed laterally across the knee joints to synchronously record knee joint range of motion.

The self-adhesive electrodes for tSCS were (5 × 5 cm Axelgard, Fallbrook, CA, United States), placed on the midline, the cathode at T10/11 and the anode at T12/L1. Electrode placement was confirmed using single pulses (monophasic, 1 ms pulse width) applied using a Digitimer DS8R Constant Current Stimulator (Digitimer, Welwyn Garden City, Hertfordshire, UK), driven by Signal software (Cambridge Electronic Design, Cambridge, UK), to test for PRR threshold in lower limb muscles. Stimulation current (mA) was increased until PRRs were observed in all measured lower limb muscles, which was defined as motor threshold. Paired pulses at an interstimulus interval (ISI) of 30 ms were then used to test the presence of post-activation depression in order to confirm activation of afferent roots. Previous studies have reported post-activation depression with tSCS paired pulses applied at ISIs between 25 and 50 ms in people with SCI [25] and in able-bodied subjects [37]. PRRs due to single and paired tSCS pulses are shown in Figure 2a. Optimal electrode location was recorded for each participant and replicated during training for participants in the STIM group. During training, tSCS was fixed at 30 Hz (biphasic, 1 ms pulse width) and applied below motor threshold (did not elicit any visible muscle contractions), at a level that induced paraesthesia in lower limb dermatomes, or the maximum level tolerated by the participant (whichever was lower), using the Chattanooga Intelect Mobile stimulator (Chattanooga Group International, Chattanooga, TN, USA). Stimulation intensity was initially established at the start of each session, and modified throughout each session as required, due to habituation to the stimulation. Transcutaneous SCS was applied tonically during each sit-to-stand manoeuvre. Stimulation was present during transitions from sit-to-stand and sit-from-stand and maintained throughout standing. No stimulation was applied during rest periods between stands.

### 2.5. Outcome Measures

#### 2.5.1. Baseline and Follow-Up Measures

Baseline and follow-up outcome measures were completed before and after the 8-week training/stimulation intervention. The International Standards for Neurological Classification of Spinal Cord Injury (ISNCSCI) assessment (without the anorectal exam) was used to observe changes in 10 myotomes on both sides of the body at baseline and final assessments. The Brain Motor Control Assessment (BMCA) was used to characterise previously undetected motor responses, from multi-muscle lower limb electromyography (EMG). A neurophysiological protocol was developed from the original BMCA procedure [38]. Recordings were performed at baseline and final assessments (experimental set-up is shown in Figure 2b). Pairs of surface 35 × 52 mm Ag-AgCl electromyography (EMG) electrodes (Covidien, Watford, UK) were placed bilaterally over the Quadriceps (Qu), Hamstring (Ham), Tibialis Anterior (TA) and Gastrocnemius (GS) muscles of the participant’s lower limbs, according to SENIAM guidelines [38]. EMG data were amplified (×1000) using a Digitimer Isolated Patient preamplifier/amplifier system (D360 8-channel Patient Amplifier System, Digitimer, Welwyn Garden City, Hertfordshire, UK) digitised at 2 kHz (Power 1401, Cambridge Electronic Design, Cambridge, UK). The data were then filtered with a pass-band of 10–200 Hz and stored on a personal computer for analysis. Additional synchronisation of bilateral electro-goniometers (DLK800, Biometrics Ltd., Wales, UK) recorded the range of movement (ROM) of knee joints. Participants lying supine, were asked to perform two bilateral voluntary manoeuvres: hip-knee flexion/extension and ankle dorsiflexion/plantar-flexion. All manoeuvres were repeated three times, cued by a light and audible tone. Health-related quality of life was assessed using the SF-36 Health Survey [39], which has been shown to be discriminative in the SCI population [40]. The SF-36 assesses 8 health-related domains: physical functioning; physical role limitations; emotional role functioning; vitality; mental health; social functioning; bodily pain; general health perception. Functional independence was assessed using the Spinal Cord Independence Measure (SCIM III) [41,42], which assesses functional status in 3 sub-categories: self-care, respiration and sphincter management and mobility.

#### 2.5.2. Weekly Measures

**Figure 3:**
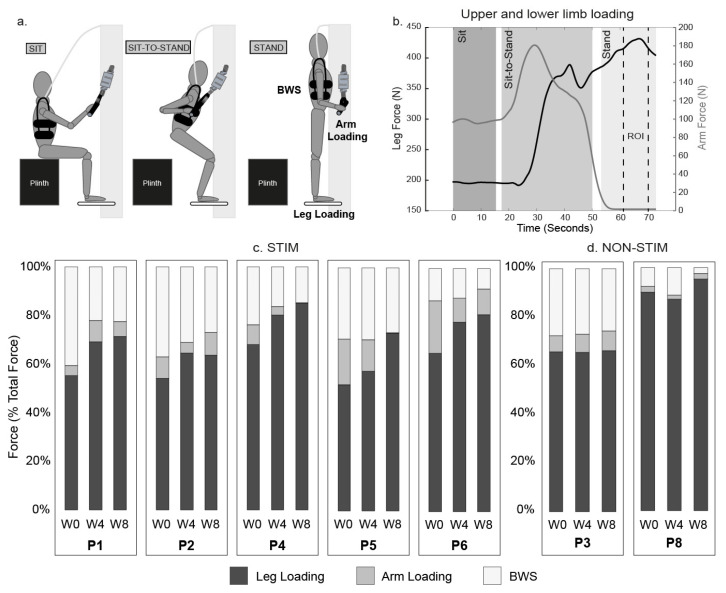
Maximum loading through the lower and upper limbs during standing. Experimental set up (**a**) and example plot of measured leg force (black) and arm force (grey) from one participant with dashed lines indicating an isolated region of interest (ROI) used to quantify force as described in 2.6 (**b**). The weekly changes in distribution of forces (measured from the ROI) are shown in (**c**,**d**). Loading though lower limbs (black), upper limbs (grey) and manually recorded bodyweight support (white) at week 0, 4 and 8 of the intervention, for all participants.

At 0, 4 and 8 weeks of training, weekly outcome measures of BWS and upper- and lower-limb loading were recorded. During sit-to-stand, the participant’s feet were positioned on a force platform (Wii Balance Board, Nintendo Co Ltd., Kyoto, Japan) to record leg loading, with their hands grasping handles (each fixed to a customised S-type load cell which measured the vertical force) to record arm loading, used for additional balance, and to lift their body weight (Figure 3). Body weight support was manually recorded from an output screen of the pneumatic resistance system.

### 2.6. Data Analysis

Vertical ground reaction force was calculated for weeks 0, 4 and 8 from the sum of the forces measured by the load cells at the four corners of the Wii Boards. Upper limb arm loading was measured for left and right handle load cells, previously calibrated with known weights. Analogue signals were processed using a moving average (2.5 s sliding window) and the maximum load on the feet during standing at weeks 0, 4, and 8 was extracted from an isolated region of interest (ROI). This ROI, a time window of <1 min, was selected based on review of video footage to isolate a period during the training session where static standing is present and not during any exercises outlined in Section 2.3. This ROI produces a less variable period for biomechanical measures to be examined.

To assess changes in muscle activation patterns between baseline and final assessments compared to published patterns of neurologically intact subjects [38], EMG envelopes (sEMG) were produced using a root mean square algorithm (0.4 s sliding window) for both flexion and extension phases for each manoeuvre (6 total). Integrated EMG (IEMG) was then calculated to express the cumulative area under the curve over time for each phase (flexion/extension) of each manoeuvre, and this was averaged across the 3 repeat trials. Simultaneous knee joint range of motion (ROM) for the flexion phase was quantified by subtracting the angle (°) at rest from the maximum angle during the flexion. The subsequent ROM during extension was quantified by subtracting the maximum flexion angle from the minimum angle in the extension phase.

SF-36 and SCIM scores for each participant were considered significant if a change greater than the Minimal Detectable Change (MDC) for each sub-category was found. For SF-36, MDCs were calculated from standard deviations reported in each sub-category from a cohort of 187 people with SCI [43]. These were Physical Functioning = 59.4, Role Physical = 40.8, Role Emotional = 11.3, Vitality = 12.7, Mental Health = 20.6, Social Functioning = 16.3, Pain = 20.6 and General Health = 21.8 For SCIM, reported MDCs were used [44]; these were Self-care = 2.6, Respiration and Sphincter Management = 6.1 and Mobility = 3.6.

## 3. Results

### 3.1. Intervention

Of the nine participants who underwent baseline measures, seven completed the 8-week intervention, and attended all training sessions. One participant experienced symptoms of autonomic dysreflexia in the evening following a training session and was later found to have an injury to the left calf. The participant was withdrawn from participating in any further training, and the incident was reported to the trial sponsor. No further adverse events were reported. Stimulation thresholds found to elicit PRRs at baseline are shown in Table 2 with the subsequent current amplitude used during training ranging between 40 and 110 mA in all participants. Paraesthesia was experienced by all participants in the STIM group during training, and tSCS at this intensity was tolerated in all participants, however some reported discomfort due to the tSCS current. In particular, when tSCS was provided at higher intensities (>80 mA), the activation of trunk muscles caused an anterior pelvic tilt in some participants, which placed strain on the spine during standing, their stimulation intensities were reduced.

During training, BWS was provided at the minimum level required to achieve standing for each participant, and was adjusted throughout the 8-week intervention, with the aim of progressively reducing the level of BWS. The set-up and an example plot of the upper and lower limb forces, recorded during a single sit-to-stand, are shown in Figure 3a,b. For each participant, peak upper and lower limb forces and BWS during standing at the first, fourth and eighth week of training is shown in Figure 3c,d.

For all participants in the STIM group, loading through the lower limbs increased progressively throughout the intervention, which was due to reduced BWS and/or reduced lifting through the upper limbs. In the NON-STIM group, neither participant showed any change in BWS with training; however, P8 had high initial leg loading, with little scope for improvement. Participants in the STIM group also reported enhanced voluntary control (ability to “actively engage” in the standing) and proprioceptive (sensory) feedback during tSCS standing activities; these effects developed over several weeks and were evident in all participants in the STIM group by Week 5. P2 and P4 regained the ability to voluntarily activate knee flexor and extensor muscles on-command in the presence of tSCS combined with standing (see P2 (AIS A) in Video S1); this occurred after 5 weeks of training. After 8 weeks of training, P4 (AIS C) regained the ability to stand with a standing frame and minimal assistance, but only in the presence of tSCS.

### 3.2. ISNCSCI Motor Scores

For each participant, ISNCSCI motor scores before and after the intervention are shown in Table 3. Lower limb motor scores increased in three of the five participants in the STIM group (+1 (AIS A), +5 (AIS C) and +7 (AIS D)) and were unchanged in the other two (both AIS A). P1 presented palpable trace function in his Achilles tendon during ankle plantarflexion in the gravity-eliminated position. P4 showed palpable trace function in her hip flexors, ankle dorsiflexors, long toe extensors and ankle plantar flexors which were absent at baseline. P5 was able to perform full ROM of his ankle dorsiflexors and knee extensors in addition to full ROM of his ankle plantar flexors in the gravity-eliminated position and was able to resist moderate pressure whilst sustaining full ROM of the long toe extensors. Lower limb motor scores were unchanged in both participants in the NON-STIM group (AIS A and AIS C). Upper limb motor scores were unchanged in all participants except for a reduction of one point recorded in one participant in the NON-STIM group.

### 3.3. Brain Motor Control Assessment (BMCA)

Before and after the intervention, participants were requested to perform voluntary hip/knee and ankle flexion and extension movements to audible cues, whilst lying supine on a couch; no tSCS was provided. Normative data in non-injured subjects demonstrates rapid and sustained recruitment of motor units in the prime mover (agonist) muscles and smaller residual amplitude response in antagonist muscles during voluntary motor tasks [38]. In SCI individuals, patterns of muscle activity range from no spinal motor output (paralysis) to appropriately sequenced reciprocal activation during controlled joint movements [38,45,46].

#### 3.3.1. Hip/Knee Flexion and Extension

**Figure 4:**
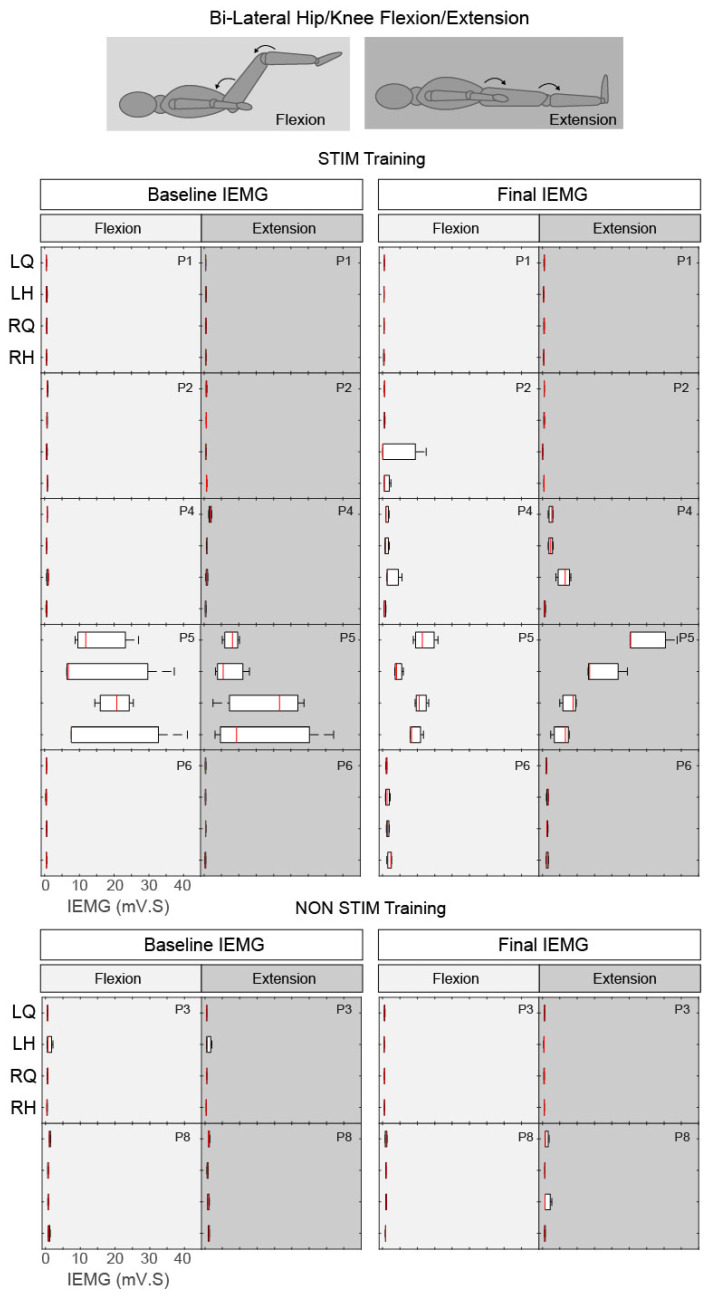
Box plots of integrated EMG activity recorded from the left (L) and right (R) quadriceps (Q) and hamstrings (H) during hip/knee flexion (light grey) and extension (dark grey) movements without tSCS, before (Baseline) and after (Final) the intervention, for all participants. Patterns of integrated EMG (IEMG) (averaged over 3 trials) are displayed for each participant during both flexion and extension phases. Each plot indicates the 25–75th percentiles (box), minimum and maximum values (whiskers), and median value (central red line).

**Figure 5:**
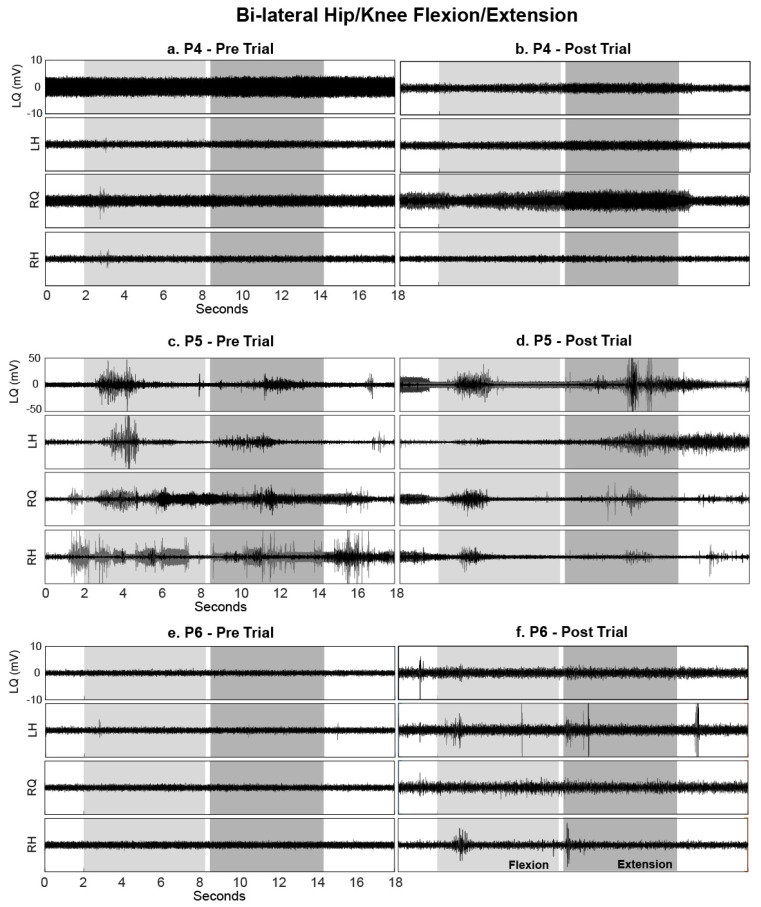
EMG activity recorded from the left (L) and right (R) quadriceps (Q) and hamstrings (H) during hip/knee flexion (light grey) and extension (dark grey) movements without tSCS, before and after the intervention. Data are shown for (**a**,**b**) P4, (**c**,**d**) P5 and (**e**,**f**) P6 (all in the STIM group); each movement was repeated three times, and EMG data from all three movements are overlaid.

During the voluntary hip/knee manoeuvre, Range of Motion (ROM) at the knee joint was unchanged at the onset of the cue, in all but one participant (P5), indicating that these participants were unable to perform the movements both before and after the intervention (all measured ROMs were <2°). P5 (AIS D, STIM group) was able to reliably perform hip/knee flexion but not extension before the intervention; uncontrolled coactivation of agonist and antagonist muscles was observed during both phases. Although coactivation was still present after training, a more appropriate activation pattern was observed, permitting the ability to extend his left lower limb from flexed (P5 left ROM extension increased from 34.4° to 98.3°). This was supported by the left quadriceps EMG activity increasing considerably during hip flexion (see P5 LQuad in Figure 4 and Figure 5) followed by an increased and sustained contraction in the left hamstrings during extension (see P5 LHam in Figure 4 and Figure 5). Although no change in knee ROM was detected, increases in EMG activity were also noted in P4 (AIS C, STIM group) and P6 (AIS A, STIM group) post training compared with pre (see P4 RQuad during extension in Figure 4 and Figure 5; P6 LHam and RHam during flexion in Figure 5). Despite this activity being present, the activation pattern was inappropriate for the phase of the task.

#### 3.3.2. Ankle Flexion and Extension

**Figure 6:**
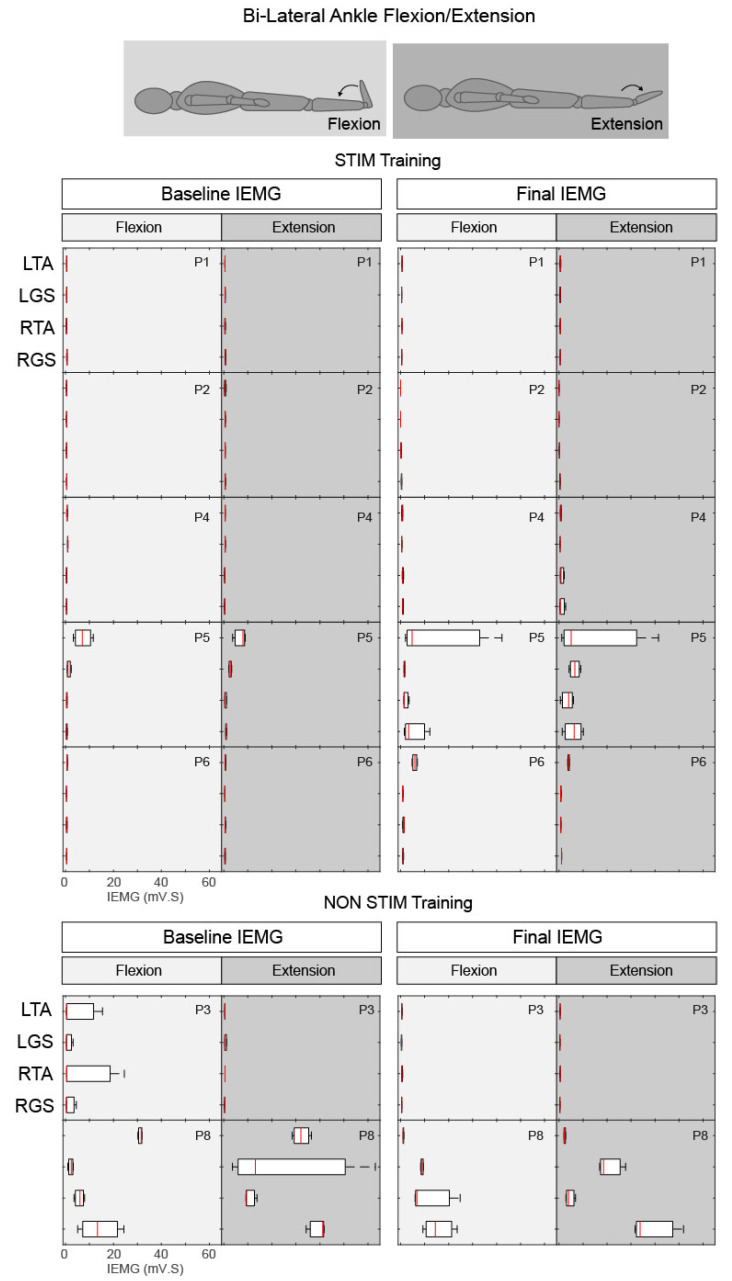
Box plots of integrated EMG activity recorded from the left (L) and right (R) Tibialis Anterior (TA) and Gastrocnemius (GS) during ankle flexion (light grey) and extension (dark grey) movements without tSCS, before (Baseline) and after (Final) the intervention for all participants. Patterns of IEMG (averaged over 3 trials) are displayed for each participant during both flexion and extension phases. Each plot indicates the 25–75th percentiles (box), minimum and maximum values (whiskers), and median value (central red line).

**Figure 7:**
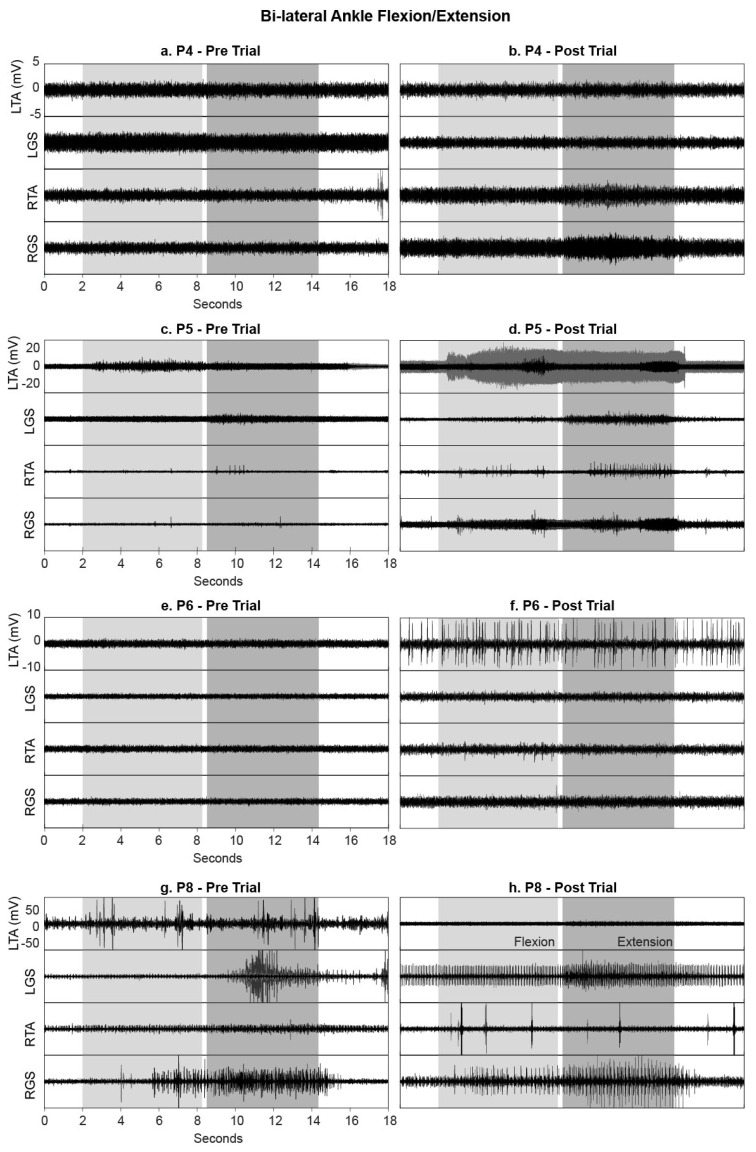
EMG activity recorded from the left (L) and right (R) Tibialis Anterior (TA) and Gastrocnemius (GS) during ankle flexion (light grey) and extension (dark grey) movements without tSCS, before and after the intervention. Data are shown for (**a**,**b**) P4, (**c**,**d**) P5, (**e**,**f**) P6 (STIM group) and (**g**,**h**) P8 (NON-STIM group); each movement was repeated three times, and EMG data from all three movements are overlaid.

During the voluntary ankle manoeuvre, participants were asked to bring their toes up and point their toes down in response to the audio/visual cue (Figure 6). P5 (AIS D, STIM group) was unable to perform both flexion and extension of the right ankle before the intervention (Figure 7c) but after training, he regained the ability (with a delayed recruitment) to flex and extend the right foot (Figure 7c,d). The EMG quantification revealed that although this functional movement was achieved, the pattern of activity was seemingly uncoordinated (Figure 6). P5 displayed an additional increase in volitional muscle activity with control over the left ankle movement in comparison to baseline recordings (Figure 7c,d). An increase in volitional EMG activity was also present in P4 (AIS C, STIM group) post training compared with pre (see P4 RTA and RGS during extension in Figure 6 and Figure 7a,b) showing coactivation of both agonist and antagonist muscles during the extension phase. Both P6 (AIS A, STIM group) and P8 (AIS C NON-STIM group) displayed increased tonic firing (see P6 LTA Figure 7e,f; P8 LGS and RGS Figure 7g,h) post training compared with pre.

### 3.4. Health-Related Quality of Life Questionnaires

**Figure 8:**
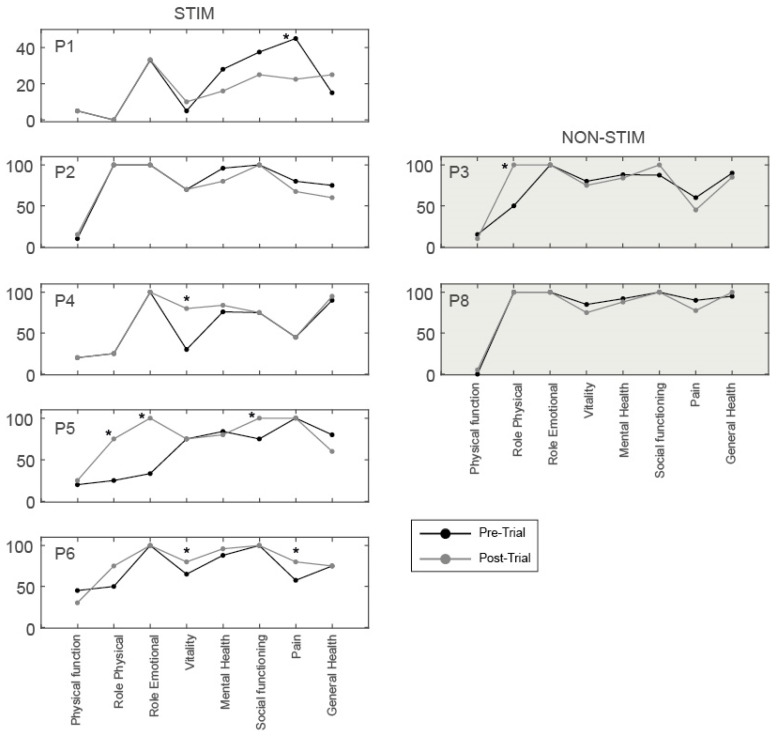
SF-36 scores across 8 sub-categories before (black) and after (grey) the intervention for participants in STIM and NON-STIM groups. * denotes a pre-post intervention change of >MDC for each participant within each sub-category [43].

**Figure 9:**
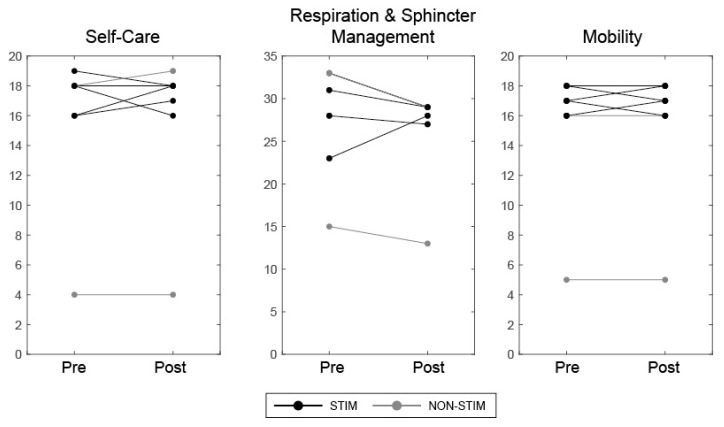
SCIM scores across three sub-categories before (pre) and after (post) the intervention for participants in STIM (black) and NON-STIM (grey) groups.

All participants in the STIM group, who had increased lower limb motor scores after training, showed improvements >MDC [43], in at least one sub-category (Figure 8). P5 showed increases in three sub-categories: Role limitations due to physical health (+50), Role limitations due to emotional problems (+67) and Social functioning (+25). P4 increased in one sub-category: Vitality (+33), and P6 increased in two sub-categories: Vitality (+15) and Pain (+23). In contrast, one participant in the STIM group (P1) showed a reduction in one sub-category: Pain (−23; i.e., the participant reported increased pain after training). In the NON-STIM participants, one participant (P3) showed a change >MDC in one sub-category: Role limitations due to physical health (+50). No changes in SCIM III scores, greater than the MDC for each sub-category [44], were noted in any participant (Figure 9).

## 4. Discussion

The aim of this pilot study was to assess the feasibility of adding sub-threshold transcutaneous SCS to an 8-week sit-to-stand training intervention in individuals with chronic motor complete and incomplete SCI. Of the 35 participants assessed for eligibility, 9 participated, and 7 completed the trial. For all participants in the STIM (tSCS + sit-to-stand training) group, loading through the lower limbs increased progressively throughout the intervention. Both participants with motor incomplete injuries in the STIM group considerably improved their ISNCSCI motor scores (by 5–7 points) and voluntary muscle activation (during the BMCA), when tSCS was switched off. Two participants in the STIM group with motor complete injuries showed small increases in volitional muscle activation in the BMCA, with tSCS switched off, and one of these participants also improved their ISNCSCI motor score by one point. No changes were found in either participant in the NON-STIM (sit-to-stand training alone) group (AIS A and C). Improvements in health-related quality of life were detected in SF-36 subcategories including physical, emotional, vitality, social functioning and pain in STIM group participants.

### 4.1. Feasibility of Study Design

There was considerable interest in the trial from people with SCI worldwide. In total, 35 potential participants were assessed for eligibility. The majority (*n* = 28) met the study criteria. The known reasons for not participating once deemed eligible were distance to travel, and assignment to the NON-STIM group. To mitigate these drop-outs, a larger trial should be carried out across multiple sites, and consider adding “sham” tSCS to the sit-to-stand alone intervention. The sensation caused by tSCS often precludes a suitable sham intervention (i.e., participants would be aware of their group assignment); however, it may be possible to inform participants that they will receive a tSCS intervention that is delivered either above or below sensory threshold. Of the nine participants that carried out the intervention, none voluntarily withdrew (two participants were withdrawn for other reasons), indicating that they found the intervention acceptable. Some participants (in the STIM group) also reported that they would be willing to continue beyond 8 weeks, indicating that a longer intervention period may also be feasible if a suitable sham intervention can be incorporated. The outcome measures were feasible overall, however stimulation artefacts caused by the tSCS precluded use of the EMG data collected during training: this issue should be further explored prior to completing a larger trial. In addition, increased pain after training was reported in one participant in the STIM group, therefore more detailed reporting of these changes during the intervention is important to distinguish the source (i.e., do they relate to the physical activity or the tSCS *per se*). Up-to 1.5 years post-trial, participants (continuing active training regimes) anecdotally reported the development of further functional changes. This highlights the importance of including long-term follow-up assessments within the study design.

### 4.2. Recovery of Motor Control in the Presence of tSCS in the STIM Group

None of our participants were able to achieve immediate unassisted or minimally assisted standing during the first session with tSCS, as has been reported by other groups [14]. This was most likely due to the tSCS being delivered at an intensity below motor threshold, so the stimulation did not directly elicit lower limb extension movements or EMG activity. However, all the participants in the STIM group showed some evidence of motor recovery in the presence of tSCS during training, which occurred after at least 4 weeks of training. This included progressive reductions in the required BWS to stand up (Figure 3c), and restored ability to voluntarily activate the quadriceps to generate lower limb extension on-command during training exercises (see Video S1). One participant (P4, AIS C) also regained the ability to stand with a standing frame with minimal assistance after 8 weeks, which was only possible in the presence of tSCS. The combination of tSCS with proprioceptive feedback, due to task-specific stand training and progressive increases in lower limb loading, may have enhanced appropriate muscle activity to enable minimally assisted standing [47].

Similar progressive recovery has previously been reported in the presence of either epidural [16,17,19,31] or transcutaneous [14,35] SCS, but recovery is dependent on preserved functional neurons passing the lesion site, even in people diagnosed with motor complete injuries [45,48]. Of the participants in the STIM group with a clinically complete diagnosis (P1, P2 and P6), none showed evidence of volitional EMG activity at baseline, however P6 was known to have retained partial innervation below the level of injury [49,50] and the other two presumably had “discomplete” injuries. It has been hypothesised that tonic SCS shifts the baseline level of spinal network excitability closer to motor threshold enabling people with incomplete and discomplete SCI to voluntarily generate movements by descending input via these preserved functional neurons [35,51].

### 4.3. Recovery of Volitional Motor Control (in the Absence of tSCS)

Improvements in voluntary control of movement or an increase in volitional muscle activity after training (when tSCS was off) were found in four of the five participants in the STIM group (P1-AIS A, P4-AIS C, P5-AIS D and P6-AIS A with partial preservation). Only one other study has reported functional recovery (with tSCS switched off) following an intervention of non-invasive SCS [35]; that study used gravity-neutral step training combined with sub-motor threshold tSCS, delivered with a 10 kHz carrier frequency. No changes were observed in either participant in our NON-STIM group (P3-AIS A and P8-AIS C), indicating the importance of the additional tSCS for these changes to occur.

The participant in the STIM group with the least severe injury (P5-AIS D), and the only participant in our study who had any evidence of voluntary motor control at baseline, recovered considerable motor function after training. He was able to fully control flexion and extension in his left leg, which was absent at baseline, and regained the ability to freely move his right foot; his ISNCSCI motor scores had improved by a total of seven points after training. Similar functional recovery (with SCS off) has previously been reported in four people with incomplete injuries (ISNCSCI sores improved by between 4 and 11 points) following intensive activity-based training combined with epidural SCS, applied at an intensity that enabled voluntarily-driven movements [33]. The other two participants with incomplete neurological diagnosis in our study had more extensive motor deficits (both showed little/no voluntary EMG activity at baseline); one was in the STIM group (P4-AIS C) and the other was in the NON-STIM group (P8-AIS C). After training, P4 (STIM) showed increases in appropriate muscle activity during the BMCA, and her ISNCSCI score improved by five points; no changes were observed in P8 (NON-STIM), indicating the importance of additional tSCS.

Among the three participants with motor complete injuries in the STIM group (P1, P2 and P6), some recovery of motor control (with tSCS off) was observed in two of them (P1 and P6). P6 had increased voluntarily driven muscle activity (during the BMCA) after training, and P1 improved his ISNCSCI motor score by one point (palpable contraction in Achilles tendon). No recovery (with tSCS off) was observed in P2, despite evidence of voluntary motor performance in the presence of tSCS during training sessions (see Video S1). Previous studies using epidural SCS in people with motor complete SCI have also reported considerable improvements in motor control (in the presence of SCS) in all participants (*n* = 12) [19,32,34], but recovery of volitional motor control with SCS off was only observed in a subset of these (5/12). Pre-clinical trials, using epidural SCS combined with step training, have also reported that animals with severe complete transections only show detectable improvements in stepping when stimulation is on, whereas animals with less severe injuries and greater lesion sparing were able to recover voluntary motor control when stimulation was absent [28,29,30,52]. Overall, in our STIM participants, the amount of recovery that occurred was related to the severity of the injury; as the progressive reduction in BWS had not reached a plateau after 8 weeks, a longer intervention may bring about further recovery.

One factor thought to be important in the effectiveness of SCS is the baseline level of excitatory support from supraspinal and peripheral systems on lumbar spinal circuity [53]: SCI disrupts this process, causing an imbalance in descending and ascending transmission and dysregulated spinal activity [4,54]. SCS had been shown to regulate this with better prognosis for those with underlying excitability at baseline, for example, in spastic conditions [28,55]. Indeed, one recent study reported that the recovery of volitional control (with SCS off), observed after one-month of epidural SCS, was correlated with spasticity scores at baseline [34]; those authors proposed that baseline spasticity might be a marker for preserved corticospinal tract axons [56]. Given that tonic sub-threshold SCS has been reported to attenuate neural hyper-excitability and recover spinal inhibitory control in people with SCI [25,26,57], and has been successfully used in the treatment of spasticity [58,59], it is also possible that acute reductions in spasticity (due to SCS) enabled the participants access to these retained pathways, which were otherwise masked by hyper-excitability in the central nervous system. Repeated activation of these pathways by carrying out sit-to-stand training in combination with SCS, over several weeks, may have contributed to the observed improvements in voluntary function without tSCS [4].

### 4.4. Stimulation Parameters

Previous studies have reported optimal parameters to elicit lower limb extension movements or standing as being at 5–15 Hz [5,6,7]. We chose a relatively higher stimulation frequency (30 Hz), which is closer to the frequency shown to suppress lower limb spasticity when applied at sub-threshold intensities [25,26,27]. Relatively higher frequencies (25–30 Hz) have also been found to be as effective for standing as 15 Hz tonic stimulation [17,60]. Another study reported that higher stimulation frequencies (80–100 Hz) augmented the activity of lower limb flexor muscles specifically, whereas lower frequencies (20–30 Hz) augmented extensor muscle activity [33]. Guidelines reported by Rejc and co-workers [60] recommend an initial stimulation frequency of 25 Hz at near-motor threshold (that does not directly elicit lower limb movements) to enable standing without BWS, however further investigation into the optimal frequency for standing is warranted, including patient-specific customisation approaches.

In this study, we used sub-threshold SCS in order to alter baseline excitability of the spinal cord, enabling movements triggered by descending inputs that remain intact after SCI [54]. While lower limb extension and standing have been directly elicited by both epidural [6] and transcutaneous [13,14] supra-threshold SCS, such high stimulation intensities have been found to cause, in some people, pulsatile contractions or rhythmic bursting that interfered with standing [60,61], and these effects may be augmented during activities in which body position is altered such as sit-to-stand, causing inconsistency in the structures being activated during movement [15,62,63]. Some of our participants also reported discomfort with higher stimulation intensities and, in some cases, co-activation of the trunk musculature caused discomfort or poor postural alignment. Therefore, to permit adequate free range of movement during stand exercises and prevent discomfort, lower stimulation intensities were selected in this trial.

### 4.5. Health-Related Quality of Life and Functional Independence

The SF-36 and SCIM III questionnaires were completed before and after training in order to explore whether any recovery of motor control was associated with improvements in healthy-related quality of life and functional independence. We used MDCs as a threshold to determine whether or not changes took place in each participant. All participants that showed evidence of functional changes after training (P4, P5 and P6) also had increased SF-36 in at least one sub-category, indicating that the motor recovery may have improved their health-related quality of life. In the pain sub-category, there were contradictory findings in the STIM group: one participant reported an improvement (P6), and one reported more pain after training (P1). This requires further investigation, including the source of any increases in pain. One participant in the NON-STIM group reported an improvement in one category (role limitations due to physical health), suggesting that the changes may have been associated with the sit-to-stand training alone, or may have occurred with any intervention provided in addition to their usual activities.

### 4.6. Limitations

The main limitation in the present study was the imbalance of participants between groups. Our intention was to recruit the same number of participants with a motor complete and motor incomplete injury in each group; which we did still achieve, however three of the five participants in the NON-STIM group were unable to complete the trial. In the STIM group participants, tSCS intensity may not have always been optimal during training, due to discomfort from the stimulation. While this cannot be avoided when using traditional tSCS waveforms, PRRs should be elicited during training to enable stimulation intensity to be defined relative to PRR threshold. Another limitation was stimulation artefacts in EMG data when tSCS was switched on. This meant that we were unable to quantify changes in EMG activity due to volitional drive in the presence of tSCS in out STIM group participants. Evidence of the motor recovery in the presence of tSCS can however be viewed in one participant in Video S1.

### 4.7. Clinical Implications and Future Work

This study has shown that the addition of non-invasive sub-threshold tSCS to sit-to-stand training is an acceptable intervention for people with motor complete and incomplete SCI. In this pilot work, we found that this intervention caused some recovery of volitional drive and control in people with clinically diagnosed complete and incomplete SCI; these findings should now be verified in a larger trial. This intervention is simple and could be achieved by people living with chronic SCI in their own homes i.e., using a standing frame and a commercially available stimulator. If larger trials support our early observations, the accessibility of this intervention could enable many people living with SCI to achieve progressive improvements in motor recovery in the presence of tSCS and, in some cases, neuroplastic change, which may also benefit health-related quality of life.

Future work should further explore the effects of sub-threshold SCS on corticospinal excitability, focusing on the effects of different stimulation intensities, in order to determine the optimal intensity for neuroplastic change, and to improve our understanding of the underlying mechanisms. Future work should also consider optimal rehabilitative interventions to combine with SCS. While there is ample pre-clinical evidence to suggest that SCS is more effective when combined with afferent feedback due to locomotor training [28,29,30], our study, and the recent clinical studies using epidural SCS [18,34], suggest that recovery of volitional motor control can occur when SCS is combined with simple exercises (incorporating descending volitional drive), which are cheaper and more accessible to the SCI population.

## Figures and Tables

**Table 1:** Participant demographic information.

	Age (Years)	Height (Meters)	Weight (Kg)	Sex (M/F)	Injury Level	Cause of Injury		AIS Grade	Time Since Injury	Group
P1	37	1.83	100	M	T3	Traumatic	Motor vehicle	A	1yr 6m	STIM
P2	38	1.92	83	M	T5	Traumatic	Motor vehicle	A	2 yr 2 m	STIM
P3	38	1.72	59	F	T6	Traumatic	Motor vehicle	A	8 yr 4 m	NON-STIM
P4	41	1.71	58	F	T5	Non-Traumatic	Spinal tumor	C	1 yr 5 m	STIM
P5	28	1.98	80	M	C6/7	Traumatic	Sports injury	D	1 yr 1 m	STIM
P6	40	1.59	61	F	T10	Traumatic	Fall	A *	9 yr 0 m	STIM
P7 ^W^	49	1.98	121	M	T10	Non-Traumatic	Vascular	B	2 yr 11 m	NON-STIM
P8	31	1.81	80	M	C5	Traumatic	Fall	C	7 yr 11 m	NON-STIM
P9 ^W^	69	1.75	70	M	T6	Non- Traumatic	Vascular	A	1 yr 4 m	NON-STIM

* Partial preservation; ^W^ withdrawn; yr, Years; m, Months.

**Table 2:** Participant stimulation thresholds.

	PRR Threshold (mA)	Average Weekly Stimulation Thresholds during Training (mA)								Paraesthesia during Training (Y/N)
	BL	W1	W2	W3	W4	W5	W6	W7	W8	
P1	50	68.8	75.2	66.3	68.3	65.7	59.1	67.5	75.4	Y
P2	150	70.8	64.9	63.7	61.1	69.6	60.0	71.7	71.8	Y
P3	120	-	-	-	-	-	-	-	-	-
P4	145	74.9	73.0	95.6	94.5	105.8	64.3	51.1	68.7	Y
P5	53	78.2	86.3	102.5	105.6	97.4	95.4	75.7	80.0	Y
P6	52	21.7	20.0	19.5	19.6	22.3	40.6	41.3	49.4	Y
P8	90	-	-	-	-	-	-	-	-	-

BL, Baseline.

**Table 3:** International Standard for Neurological Classification of Spinal Cord Injury (ISNCSCI) before (pre) and after (post) the intervention for participants in transcutaneous spinal cord stimulation (tSCS) combined with sit-to-stand training (STIM (S)) and sit-to-stand training alone (NON-STIM (NS)) groups.

	Group	Injury Level	AIS Grade	Upper Motor Max = 50			Lower Motor Max = 50		
				Pre	Post	Diff	Pre	Post	Diff
P1	S	T3	A	50	50	0	0	1	+1
P2	S	T5	A	50	50	0	0	0	0
P3	NS	T6	A	50	50	0	0	0	0
P4	S	T5	C	50	50	0	1	6	+5
P5	S	C6/7	D	49	49	0	24	31	+7
P6	S	T10	A	50	50	0	1	1	0
P8	NS	C5	C	20	19	−1	0	0	0

## Supplementary Materials

The following are available online at https://www.mdpi.com/2077-0383/9/9/2765/s1, Video S1: Volitional motor recovery with tSCS.
